# Optimization of the Photon Path Length Probability Density Function-Simultaneous (PPDF-S) Method and Evaluation of CO_2_ Retrieval Performance Under Dense Aerosol Conditions

**DOI:** 10.3390/s19051262

**Published:** 2019-03-12

**Authors:** Chisa Iwasaki, Ryoichi Imasu, Andrey Bril, Sergey Oshchepkov, Yukio Yoshida, Tatsuya Yokota, Vyacheslav Zakharov, Konstantin Gribanov, Nikita Rokotyan

**Affiliations:** 1 Atmosphere and Ocean Research Institute, The University of Tokyo, Kashiwa 277-8568, Japan; imasu@aori.u-tokyo.ac.jp; 2 Institute of Physics of National Academy of Sciences of Belarus, 68 Prospekt Nezavisimosti, Minsk BY-220072, Belarus; andrey.bril@gmail.com (A.B.); sergey.http@gmail.com (S.O.); 3 National Institute for Environmental Studies, Onogawa 16-2, Tsukuba 305-8506, Japan; yoshida.yukio@nies.go.jp (Y.Y.); yoko@nies.go.jp (T.Y.); 4 Laboratory of Climate and Environmental Physics, Ural Federal University, Lenina Ave. 51, Yekaterinburg 620083, Russia; v.zakharov@remotesensing.ru (V.Z.); kgribanov@remotesensing.ru (K.G.); nikita.rokotyan@urfu.ru (N.R.); 5 Institute of Mathematics and Mechanics, UB RAS, S.Kovalevskay Street, 16, Yekaterinburg 620990, Russia

**Keywords:** aerosols, carbon dioxide (CO_2_), greenhouse gases observing satellite (GOSAT), photon path length probability density function (PPDF), retrieval, short wavelength infrared (SWIR)

## Abstract

The photon path length probability density function-simultaneous (PPDF-S) algorithm is effective for retrieving column-averaged concentrations of carbon dioxide (XCO_2_) and methane (XCH_4_) from Greenhouse gases Observing Satellite (GOSAT) spectra in Short Wavelength InfraRed (SWIR). Using this method, light-path modification attributable to light reflection/scattering by atmospheric clouds/aerosols is represented by the modification of atmospheric transmittance according to PPDF parameters. We optimized PPDF parameters for a more accurate XCO_2_ retrieval under aerosol dense conditions based on simulation studies for various aerosol types and surface albedos. We found a more appropriate value of PPDF parameters referring to the vertical profile of CO_2_ concentration as a measure of a stable solution. The results show that the constraint condition of a PPDF parameter that represents the light reflectance effect by aerosols is sufficiently weak to affect XCO_2_ adversely. By optimizing the constraint, it was possible to obtain a stable solution of XCO_2_. The new optimization was applied to retrieval analysis of the GOSAT data measured in Western Siberia. First, we assumed clear sky conditions and retrieved XCO_2_ from GOSAT data obtained near Yekaterinburg in the target area. The retrieved XCO_2_ was validated through a comparison with ground-based Fourier Transform Spectrometer (FTS) measurements made at the Yekaterinburg observation site. The validation results showed that the retrieval accuracy was reasonable. Next, we applied the optimized method to dense aerosol conditions when biomass burning was active. The results demonstrated that optimization enabled retrieval, even under smoky conditions, and that the total number of retrieved data increased by about 70%. Furthermore, the results of the simulation studies and the GOSAT data analysis suggest that atmospheric aerosol types that affected CO_2_ analysis are identifiable by the PPDF parameter value. We expect that we will be able to suggest a further improved algorithm after the atmospheric aerosol types are identified.

## 1. Introduction

Among the greenhouse gases emitted by human activities, carbon dioxide (CO_2_) has the largest radiative forcing as a total effect. Its atmospheric concentration has been increasing since the advent of the Industrial Revolution. The global distribution and temporal variations of the gas have been clarified based on ground-based in situ measurements [1]. However, observation sites are unevenly distributed on a global scale, particularly over the ocean. For this reason, global measurements by satellite have long been recommended.

Based on that background, attempts have been made to retrieve CO_2_ concentration data from Thermal InfraRed (TIR) spectra observed by satellite sensors since the 1990s. Chédin et al. [2] first detected a CO_2_ signal from TIR spectra, which is sensitive to CO_2_ concentration changes in the upper troposphere measured by The High-Resolution Infrared Radiation Sounder (HIRS). Then, they applied the method to demonstrate the usage of the data for CO_2_ source/sink analysis [3]. Following these studies, many others have been conducted using sensors of similar types: the Interferometric Monitor for Greenhouse gases (IMG) [4,5], Infrared Atmospheric Sounding Interferometer (IASI) [6], Tropospheric Emissions Spectrometer (TES) [7], Atmospheric Infrared Sounder (AIRS) on the Aqua satellite [8], and Thermal And Near-infrared Sensor for carbon Observation–Fourier Transform Spectrometer (TANSO-FTS) (Band 1, 0.758–0.775 μm; Band 2, 1.56–1.72 μm; Band 3, 1.92–2.08 μm; Band 4, 5.56–14.3 μm; where Band 4 corresponds to the TIR region) on the Greenhouse gases Observing Satellite (GOSAT) [9,10].

However, TIR sounders are, in principle, sensitive to the CO_2_ concentration in the troposphere [11], but the sensitivity is low in the planetary boundary layer. This point presents important disadvantages for the study of CO_2_ surface fluxes. It is necessary to measure CO_2_ concentrations at a low altitude or a columnar concentration for CO_2_ budget analysis because most CO_2_ sources and sinks are located near the Earth’s surface.

By contrast, it is generally preferable to analyze the radiance spectra measured in Short Wavelength InfraRed (SWIR) to analyze the CO_2_ concentration rather than in TIR because the SWIR spectra are sensitive to CO_2_ concentration changes near the surface. The dry air columnar mixing ratio (XCO_2_) can be retrieved from the data. The following are examples of satellite sensors that observe spectra in SWIR to measure XCO_2_: Scanning Imaging Absorption Spectrometer for Atmospheric Chartography (SCIAMACHY) [12], TANSO-FTS (Bands 1, 2 and 3) on the GOSAT [13], Orbiting Carbon Observatory-2 (OCO2) [14], Chinese Carbon Dioxide Observation Satellite Mission (TanSat) [15], and Carbon Monitoring Satellite (CarbonSat) [16]. Although the usage of the SWIR band presents the benefit that signals from vertically integrated CO_2_ are obtainable, the spectra can be easily affected by multiple light scattering because of clouds and aerosols in the atmosphere. This effect must be examined in order to accurately retrieve XCO_2_ from SWIR.

The first satellite dedicated to observing greenhouse gases was GOSAT, which was launched in 2009 to measure greenhouse gases: CO_2_ and methane (CH_4_). Its main sensor, TANSO-FTS, observes atmospheric radiance spectra in both SWIR and TIR ranges.

Many attempts have been undertaken to develop retrieval algorithms to derive XCO_2_ from the SWIR band data. Most of these algorithms are based on the so-called ‘Full Physics (FP)’ method. One is used to process standard products of XCO_2_ released from the GOSAT project at the National Institute for Environmental Studies (NIES), which can also be classified as an FP method. In this method, the multiple light scattering processes are explicitly calculated by solving radiative transfer equations accounting for the optical properties of aerosols [17,18]. Many research groups have adopted methods of this type: NIES (Japan) [17,18], Atmospheric CO_2_ Observations from Space (ACOS) (NASA JPL, USA) [19,20], Netherlands Institute for Space Research/Karlsruhe Institute of Technology (SRON-KIT) (Netherlands and Germany) [21,22,23], University of Leicester (UK) [14,24,25], and Yonsei University (Korea) [26,27].

A more simplified estimation method, the photon path length probability density function (PPDF)-based method, has been proposed (e.g., [28,29,30,31]). This method can represent the effects of light-path modification without exact radiative transfer calculations because the light-path modification is evaluated statistically for atmospheric transmittance including clouds/aerosols using PPDF parameters. In the new version of the PPDF-based method, the PPDF-simultaneous (PPDF-S) method, gas concentrations are simultaneously retrieved with PPDF parameters [31]. Details of PPDF-based methods are described in Section 2.

Comparing XCO_2_ data retrieved using the NIES FP algorithm and those retrieved using the PPDF-S algorithm, the results show that the difference in XCO_2_ is large over the middle of Africa and Western Siberia, where many aerosols exist because of biomass burning [32]. The objective of this study is to improve the PPDF-S algorithm and to tune its parameters under high aerosol conditions, as well as to evaluate the retrieval performance of the method through simulation studies. Then, the improved PPDF-S algorithm and tuned parameters are applied to analyses of GOSAT data observed over Western Siberia during the biomass burning season. Additionally, the XCO_2_ retrieval accuracy is evaluated.

## 2. PPDF-S Retrieval Method

### 2.1. Basis of PPDF-S Retrieval

Most sensors designed to measure gaseous constituents in the atmosphere, such as TANSO-FTS onboard GOSAT, detect the solar radiation in the SWIR region reflected by the earth’s surface or scattered by the atmosphere. When we analyze CO_2_ concentrations from the measured spectral radiation, scattering effects by clouds/aerosols in the atmosphere make the analysis very difficult because they shorten and lengthen the light-path length from the geometrical length. If these properties are estimated inappropriately, then large errors in the estimation of CO_2_ concentrations can easily occur.

**Figure 1:**
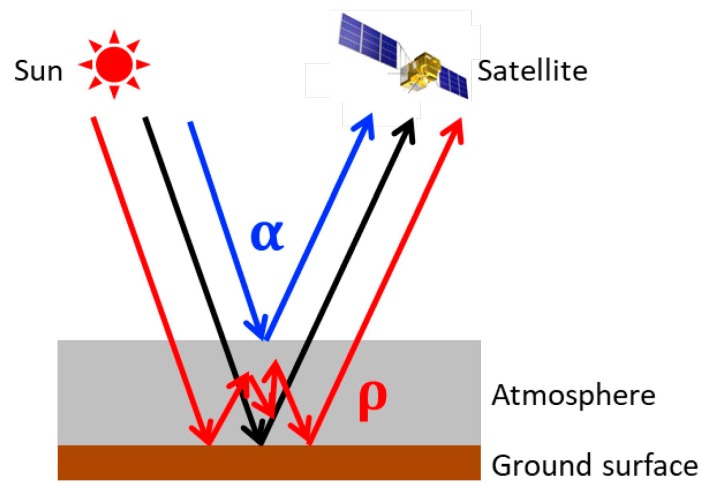
Schematic showing definitions of PPDF parameters *α* and *ρ*. The blue line shows the light-path length shortened because of light reflection, which is represented by *α*. The red line is the path length lengthened because of multiple light scattering, which is represented by *ρ*. The black line shows the basic geometrical path of light propagation.

In the FP method adopted for GOSAT data analyses, the scattering processes in the atmosphere including aerosols are represented using the Discrete Ordinate Method (DOM) for accurate XCO_2_ retrieval [17,18]. However, the PPDF-based method does not solve the radiative transfer equation directly for the representation of scattering effects. In the method, the light-path modification is described by effective transmittance using the following basic PPDF parameters: the altitude of the cloud/aerosol layer *h*; the relative reflection of the cloud/aerosol layer *α*, which represents the relative fraction of the photons scattered singly to the detector; *ρ*, which characterizes the relative mean path length between the layer and ground surface; and an adjustment parameter *γ* accounting for multiple scattering (e.g., [28,29,30,31]). A diagram of the definitions of *α* and *ρ* is presented in Figure 1.

Oshchepkov et al. [31] proposed the use of effective transmittance within a three-layered PPDF model using eight PPDF parameters to characterize the light-path modification. Four parameters represent the Rayleigh scattering effects: *h_r_*, *α_r_*, *ρ_r_*, and *γ_r_*. The remaining four parameters represent the aerosol reflection/scattering effects: *h_a_*, *α_a_*, *ρ_a_*, and *γ_a_*. Using these parameters, the effective transmittance can be represented by the following equations:

(1) $$T_{eff}=\alpha_{r}T_{3}+\left(1-\alpha_{r}\right)T_{12}^{r}T_{a}T_{3},$$

(2) $$T_{3}=\exp\left[-C_{\mu }\tau_{3}\right],$$

(3) $$T_{12}^{r}=\exp\left[-C_{\mu }\left(1+\delta_{r}\right)\tau_{12}\right],$$

(4) $$T_{a}=\left(1-\alpha_{a}\right)\exp\left[-C_{\mu }\tau_{a}\delta_{a}\right]+\alpha_{a}\exp\left[+C_{\mu }\tau_{a}\right],$$

where

(5) $$\delta_{r}=\rho_{r}\exp\left[-\gamma_{r}\tau_{12}\right],$$

(6) $$\delta_{a}=\rho_{a}\exp\left[-\gamma_{a}\tau_{a}\right],$$

(7) $$C_{\mu }=1/\cos\theta +1/\cos\theta_{0},$$

(8) $$\tau_{a}=\int_{0}^{h_{a}}k(h)dh,\;\tau_{12}=\int_{0}^{h_{r}}k(h)dh,\;\tau_{3}=\int_{h_{r}}^{h_{TOA}}k(h)dh,$$

where *θ* and *θ_0_* respectively denote the solar and satellite zenith angles; *k*(*h*) is the gas absorption coefficient at altitude *h*; and *h_TOA_* is the altitude of the top of the atmosphere.

When the gas concentrations are simultaneously retrieved with the PPDF parameters described above, the method is designated as the PPDF-simultaneous (PPDF-S) method: the newest version among PPDF-based methods applied to GOSAT measurements. The history of the development of the PPDF-S method and an explanation of its performance are described in the following section.

### 2.2. History and Performance of PPDF-S

Actually, NIES has started to develop a PPDF-based retrieval method for application to GOSAT SWIR measurements. First, based on the forward Monte Carlo simulation for the analysis of photon trajectory statistics by Bril et al. [28], four PPDF parameters were introduced: *h*, *α*, *ρ*, and *γ*. In the next step, Oshchepkov et al. [29] used PPDF parameters for XCO_2_ retrieval and established the PPDF-based retrieval method. After Bril et al. [33] improved the method for application to the atmosphere including desert dust aerosols, Oshchepkov et al. [30] executed an additional improvement defining two sets of PPDF parameters. Those parameters, *h_c_*, *α_c_*, *ρ_c_*, and *γ_c_*, and *h_a_*, *α_a_*, *ρ_a_*, and *γ_a_*, were introduced respectively to distinguish the scattering process by clouds and that by aerosols. The parameters were applied to the three-layered atmosphere. The method was used for GOSAT data processing. Global characteristics of the retrieved XCO_2_ and PPDF parameters were subsequently investigated by Oshchepkov et al. [34].

To evaluate the retrieval accuracy of the improved PPDF-based method, the retrieved XCO_2_ was validated using ground-based remote sensing data provided by the Total Column Observing Network (TCCON [35]) by Oshchepkov et al. [36]. PPDF-based retrieval results and validation results were cross-compared with those of other XCO_2_ results retrieved using various retrieval algorithms based on the FP method in Oshchepkov et al. [37]. According to Oshchepkov et al. [36], the validation results in comparison with TCCON 12 sites data showed that PPDF-based XCO_2_ had a bias of −0.43 ppm and a standard deviation of 1.80 ppm. The paper also showed that the detected effects of light-path modification represented by the PPDF parameters were physically interpreted by the seasonal trends of the aerosol optical depth derived from an aerosol transport model, as well as by the time series of cirrus optical depth derived from space-based lidar measurements. By comparing the results of these retrieval analyses of XCO_2_ of different types, Oshchepkov et al. [37] demonstrated that PPDF-based XCO_2_ was as accurate as the retrieval results obtained using other algorithms based on the FP method.

In the earlier version of the PPDF-based algorithm, the PPDF parameters were used as a pre-screening test to identify GOSAT observation data that are not distinctly affected by atmospheric light scattering. The near clear sky conditions are recognized by low values of PPDF parameters retrieved from radiance spectra in the O_2_ A-band (0.76 μm). After the prescreening test, the gas concentration retrieval is executed in the near-infrared CO_2_ bands, assuming that light-path modification is nearly zero. The retrieval scheme is based on Differential Optical Absorption Spectroscopy (DOAS). Therefore, the method is designated as a PPDF-D method. However, the observation data that are used for gas retrieval with the method are limited. For the PPDF-D method, further improvement was executed to retrieve PPDF parameters and CO_2_ concentration simultaneously, which enables an increase of the amount of observation data that are applicable for the retrieval. First, Bril et al. [38] reported pre-results of the simultaneous retrieval of XCO_2_ and PPDF parameters. From the retrieval, only two PPDF parameters were derived from GOSAT data measured over ocean areas: the effective altitude of the aerosol layer (*h_a_*) and the relative layer reflectivity (*α_a_*). Subsequently, Oshchepkov et al. [31] further improved the method for the simultaneous retrieval of XCO_2_ and eight PPDF parameters: *h_r_*, *α_r_*, *ρ_r_*, *γ_r_* for Rayleigh scattering by atmospheric molecules; and *h_a_*, *α_a_*, *ρ_a_*, and *γ_a_* for scattering by aerosol. The paper established the current PPDF-S method. The retrieved XCO_2_ was validated in comparison with TCCON data at 12 sites. Reportedly, the accuracy of the study was a bias of 0.08 ppm, with a standard deviation of 1.90 ppm.

Iwasaki et al. [32] also validated the PPDF-S-based XCO_2_, as well as XCH_4_, by expanding the time period of validation and describing their global characteristics, which demonstrated that the bias and standard deviation of XCO_2_ over the land are, respectively, 0.73 and 1.83 ppm. The paper also presented validation of the Level 2 data as standard products for General Users (i.e., GU products) and only for Research Announcement principal investigators (RA products) retrieved using the FP method and released from NIES in comparison with the PPDF-S-based products. GU products were selected from RA products using screening tests referring to atmospheric parameters, such as aerosol optical thickness (AOT), surface conditions, and radiometric parameters.

To investigate the uncertainty of the retrieved results of the GOSAT data observed under high-AOT conditions, the validation results were compared for the XCO_2_ of the PPDF-S product and the data removed from the RA products through screening tests, which were mainly removed because of the high-AOT values. From the analysis, it was pointed out that the XCO_2_ bias and its standard deviation in the PPDF-S product are smaller than those of the removed RA products.

However, comparing the global distribution of PPDF-S products to those of the GU products, large differences in both the XCO_2_ and XCH_4_ were found over the middle of Africa and Western Siberia, where biomass burning occurs and where large amounts of aerosols exist. The different retrieval performances of PPDF-S and FP methods might result from the poor representativity of the light-path modification by the PPDF-S method under high-AOT conditions. As a conclusion of this study, the importance of improving the PPDF-S retrieval algorithm to be more accurate under denser aerosol conditions is indicated. The improvement method used for this study is described in Section 3.

## 3. Methodology

### 3.1. Basic Equations of Retrieval

The PPDF-S retrieval algorithm is based on optimal estimation to minimize a cost-function ***J*(*x*)**, the weighted least-squares difference [39], which is defined by four components: measured radiance spectra R*; simulated radiance spectra ***R′***; the a priori data ***x_a_*** for the target state vector ***x***; the state vector about the amount of gases in each atmospheric layer ***x_gas_***; and other state vectors ***x_nongas_***, including *h_r_*, *α_r_*, *ρ_r_*, *γ_r_*, *h_a_*, *α_a_*, *ρ_a_*, and *γ_a_*. ***J*****(*x*)** is formatted as

(9) $$J\left(R*,x_{a},x\right)={\left(Y*-F\left(x\right)\right)}^{T}S_{y}^{-1}\left(Y*-F\left(x\right)\right)+{\left(x_{a}-x\right)}^{T}S_{a}^{-1}\left(x_{a}-x\right),$$

where

(10) $$x=\left(\begin{array}{c} x_{\mathrm{gas}}\\ x_{\mathrm{nongas}} \end{array}\right),\;S_{a}=\left(\begin{array}{cc} S_{a,\mathrm{gas}} & 0\\ 0 & S_{a,\mathrm{nongas}} \end{array}\right),$$

(11) Y*=−ln(R*), F(x)=−ln(R′)=−ln(f(x)),

and *f*(***x***) is the forward model based on PPDF parameters. Additionally, ***S_y_*** and ***S_a_*** are covariance matrices of measurement errors and a priori data.

Following the Gauss–Newton method, the solution is obtained in an iterative manner as

(12) $$x_{i+1}=x_{i}+{\left(K_{i}^{T}S_{y}^{-1}K_{i}+S_{a}^{-1}\right)}^{-1}\left[K_{i}^{T}S_{y}^{-1}\left(Y*-F\left(x_{i}\right)\right)-S_{a}^{-1}\left(x_{i}-x_{a}\right)\right],$$

where ***K_i_*** is the Jacobian matrix, calculated with respect to ***x*** = ***x_i_*** as

(13) $$K_{i}={\frac{\partial F\left(x\right)}{\partial x}|}_{x=x_{i}.}$$

Hereinafter, each element of ***x_a_*** is described as *x_a_*. The square root values of the diagonal element of ***S_y_*** and ***S_a_*** are respectively described as *σ*_y_ and *σ*_a_ for this study.

### 3.2. CO_2_ retrieval Based on Simulation

To investigate the accuracy of the PPDF-S retrieval, simulation studies have been conducted. In the analysis, measured radiance described as Y* in the equation for the cost function is simulated by an atmospheric Radiative Transfer Model (RTM), assuming various atmospheric conditions and surface characteristics. The CO_2_ amount is retrieved from the simulated radiance. The retrieval accuracy is defined in this study as the XCO_2_ bias, which is calculated by subtracting the assumed XCO_2_ in the simulation from the retrieved XCO_2_. The RTM parameter settings and datasets used for the calculation of radiance are shown in Table 1. In the calculation, a clear sky condition is also presumed by setting AOT = 0.0.

On the other hand, ***F(x)*** is calculated using PPDF parameters in the retrieval process as a forward calculation step. The solar irradiance model, zenith angles, surface albedo, and atmospheric profiles, which are used for the forward calculation, are the same as those used for the Pstar3 simulation, except for the aerosol models. In the PPDF-based forward model, light-path modification is explained as the change of effective transmittance described by PPDF parameters for any aerosol model. The a priori and variance of each parameter included in the state vector ***x*** are shown in Table 2. The values of *σ*_y_ in the retrieval are shown below.

*σ*_y_ for Band 1: 4.0 × 10^−7^/SNR [W/m^2^/str/cm^−1^] *σ*_y_ for Band 2: 3.5 × 10^−7^/SNR [W/m^2^/str/cm^−1^] *σ*_y_ for Band 3: 2.5 × 10^−7^/SNR [W/m^2^/str/cm^−1^] signal-to-noise ratio (SNR) = 400

### 3.3. Optimization of PPDF Parameter Settings for More Adequate XCO_2_ Retrieval

For the accurate retrieval of XCO_2_, it is generally important to derive a stable CO_2_ profile. To achieve this, PPDF parameters should also be derived appropriately because the state vector ***x_gas_*** for the CO_2_ amount in each layer and ***x_nongas_*** for PPDF parameters are complementary, and because an inappropriate setting of variation of the former parameters can engender instability in the CO_2_ profile.

However, it is difficult to determine the appropriate value and variance of PPDF parameters a priori because PPDF parameters must represent light reflectance/scattering situations of many types with a limited number or parameters and because radiation propagation processes are complicated. If very large values are set to ***S_a_*** when describing the wide range of dispersion a priori, then the possibility exists that an inappropriate value of retrieved PPDF parameters from correct answers will engender an undesirable value of XCO_2_.

One possible means of finding the best value or at least a value better than the current value of the variance of state vector (*σ_a_*), as well as the priori value (*x_a_*), is to find parameters with unnecessarily large values and then make them smaller than the current values. The spectral radiance corresponding to each variance can be a good measure to evaluate the effects of the variation of each PPDF parameter on XCO_2_ retrieval. The radiance can be evaluated through the radiative transfer equation ***Y*** = ***K***·*σ_a_*. Based on the idea that a larger value of ***K***·*σ_a_* more effectively influences the parameter affecting XCO_2_ retrieval, we found the PPDF parameter with unnecessarily large variance and made it smaller to optimize the parameter: we constrained the parameter to its better a priori value. We determined the more appropriate value of *σ_a_* by referring to the shape of the retrieved vertical profile of CO_2_ concentration as a measure of a stable solution. The optimization was aimed at improving the accuracy of CO_2_ concentration for various atmospheric conditions, irrespective of existence or non-existence of the aerosol or aerosol type.

## 4. Retrieval Performance Based on Simulation Studies

### 4.1. PPDF Parameter Optimization

**Figure 2:**
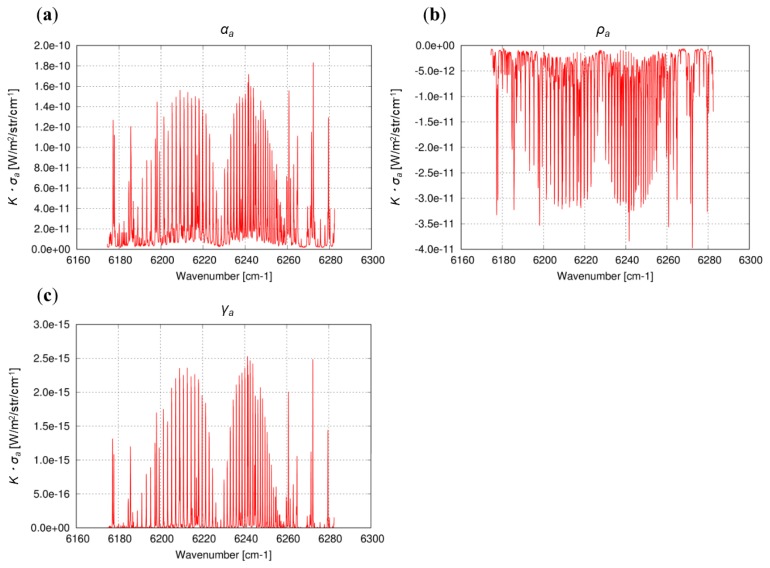
Radiance spectrum of the ***K***·*σ_a_* for *α_a_* (**a**), *ρ_a_* (**b**), and *γ_a_* (**c**) in Band 2.

We compared the value of ***K***·*σ_a_* for each PPDF parameter and evaluated the effect of its variation on XCO_2_ retrieval. The formulation of the Jacobian ***K*** for each parameter is based on the procedure reported by Oshchepkov et al. [30]. Examples of spectral ***K***·*σ_a_* for *α_a_*, *ρ_a_*, and *γ_a_* in Band 2 are shown in Figure 2. Results of comparisons showed that the ***K***·*σ_a_* of *α_a_* representing the light reflectance by aerosols is greater by about two orders of magnitude among the PPDF parameters (*α_r_*, *ρ_r_*, *γ_r_*, *ρ_a_*, and *γ_a_*) in all GOSAT measurement bands. Consequently, the variation of *α_a_* might be too large to cause instability of the retrieved CO_2_ profile. Therefore, we tried to optimize the *σ_a_* of *α_a_*, as well as its a priori value, based on the results retrieved from various simulations using the radiative transfer code, Pstar3.

As a method of optimizing the deviation value, we tested various magnification values, which were multiplied by the original value of the *σ_a_* from 1 to 1/100. Furthermore, we investigated the retrieval accuracy of XCO_2_. Referring to the shape of the retrieved CO_2_ vertical profile, the results showed that 1/20 is the most adequate magnitude for *σ_a_*. We also found that the value of 1/200 instead of 1/20 used in the equation for the a priori value of *α_a_* shown in Table 2 for *β_αa_* was effective at reducing XCO_2_ bias with the smaller *σ_a_*. The retrieval results obtained using the original settings are shown in Table 2. Results based on the optimization explained above are compared in relation to both a clear sky condition and atmosphere including aerosols of various types.

### 4.2. CO_2_ Retrieval Results

For the retrieval simulations, the true value of XCO_2_ is assumed to be 390 ppm. The a priori value of XCO_2_ is set as 385 ppm, as described in Section 3.2. ‘XCO_2_ bias’ is defined in this subsection as the value resulting from subtraction of the true XCO_2_ of 390 ppm from the retrieved XCO_2_.

#### 4.2.1. Clear Sky Condition

**Figure 3:**
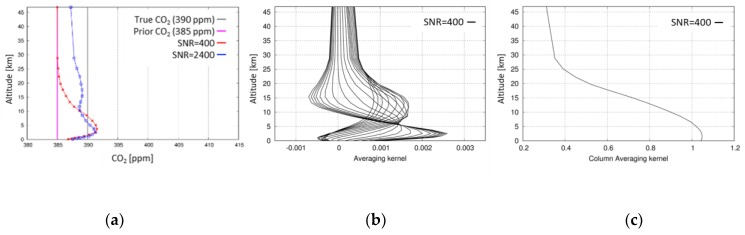
Vertical CO_2_ profile (**a**), averaging kernel (**b**), and column averaging kernel (**c**) retrieved by the optimized method.

**Figure 4:**
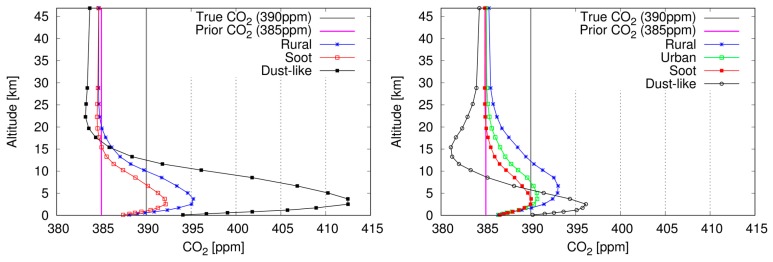
CO_2_ profiles retrieved using the original method (**left**) and the optimized method (**right**) assuming AOT of 1.0 at 0.55 μm for the atmosphere, including Rural (blue), Urban (green), Soot (red), and Dust-like (black) aerosol types. Surface albedos are 0.2 for all GOSAT measurement bands.

For synthesizing the measured radiance under a clear sky condition, surface albedo is assumed to be 0.2 for all spectral bands of the GOSAT sensor. The averaging kernel which represents the vertical resolution of the retrieval analysis and the column averaging kernel which represents the sensitivity profile to the total column value of the retrieved parameter are almost identical when comparing the original method and the optimized method for various atmospheric conditions. As examples, the vertical profile of the retrieved CO_2_ concentration, averaging kernel, and column averaging kernel are shown in Figure 3a–c. The scale on the horizontal axis of Figure 3a is set to be the same as that of the results for the atmosphere including aerosols, as depicted in Figure 4. In Figure 3a, the retrieved CO_2_ profiles assuming SNR = 400 and 2400 are depicted by red and blue lines, respectively. The vertically constant profile of 385 ppm is the a priori profile (pink line) and that of 390 ppm is the true profile (gray line) in the figure. The retrieved CO_2_ profile for SNR = 400 exists between the a priori and true profiles, but its shape is not vertically uniform, with a peak at a height in the middle of the troposphere corresponding to the sensitivity peak of the retrieval system. If the SNR of the measurement was ideally very high, for example, 2400, then the retrieved profile was closer to the true profile, but it remained not perfectly uniform. This point of caution is discussed in Section 6. Figure 3b,c present the results obtained when assuming SNR = 400.

The XCO_2_ biases of the results retrieved using the original method and the optimized method are −0.47 ppm and −0.63 ppm, respectively. The accuracy of XCO_2_ becomes slightly worse for the clear sky condition after the optimization of *α_a_*, but the value is still sufficiently small compared to the variation in CO_2_ concentration of natural phenomena.

#### 4.2.2. Atmosphere Including Aerosols of Various Types

In the simulation for the atmosphere including aerosols, the measured radiance is synthesized assuming various values of surface albedo, aerosol types, and AOT, as shown in Table 1. In Figure 4, examples show the CO_2_ profile retrieved using the original method (left) and the optimized method (right) in the cases of AOT of 1.0 at 0.55 μm for aerosols of four types: Rural (blue), Urban (green), Soot (red), and Dust-like (black). For these calculations, the surface albedos are set as 0.2 for all GOSAT spectral bands. Because the averaging kernel and column averaging kernel are almost identical to those under the clear sky condition, they are not shown here.

In the original method, the retrieval results obtained under the condition that includes Urban-type aerosols are not provided in the figure because the solution related to ***x*** does not converge on a value in the retrieval iteration analysis based on a Gauss–Newton method. However, the retrieval is completed to obtain solutions by the optimized method of all conditions. Although the variance of the CO_2_ profile retrieved by the optimized method for Dust-like aerosols becomes smaller after the optimization of α_a_, it is still larger than those found for aerosols of other types.

XCO_2_ biases for the aerosols of four types are evaluated as explained below.

- Rural: +1.31 ppm (original method), +0.48 ppm (optimized method)
- Urban: not converged (original method), −1.27 ppm (optimized method)
- Soot: −0.95 ppm (original method), −2.03 ppm (optimized method)
- Dust-like: +11.38 ppm (original method), −1.22 ppm (optimized method)

**Figure 5:**
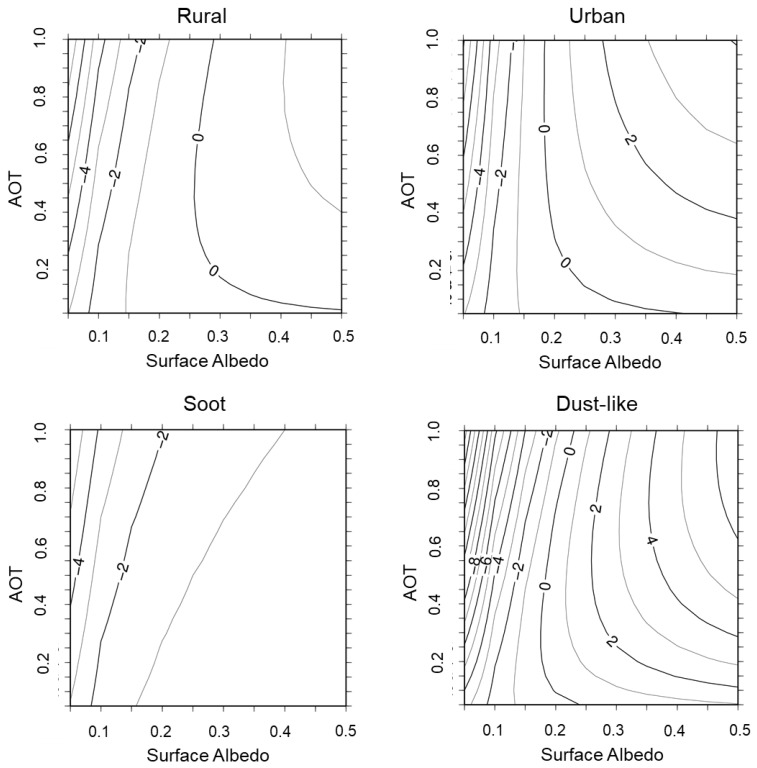
XCO_2_ biases calculated for various combinations of surface albedo and AOT. Units are parts per million.

For synthetic analyses of the retrieval accuracy after the optimization for *α_a_*, XCO_2_ biases are calculated for various combinations of surface albedo and AOT. A contour plot of the results is portrayed in Figure 5. They show that the bias (in parts per million: ppm) depends on the aerosol type, surface albedo, and AOT. Especially when the surface albedo is small, the dependence of the XCO_2_ bias on AOT is large for aerosols of all types. Each result has a line representing zero bias, except for Soot, which shows a negative bias in all cases. For Dust-like aerosols, the bias exceeds ±5 ppm. It is larger than those for other types of aerosols. Urban aerosols comprise Rural and Soot aerosols. Therefore, the XCO_2_ bias for the Urban type apparently has an intermediate property of those for the Rural and Soot aerosols.

## 5. Application of the Optimized Method to GOSAT Data Observed for Western Siberia

As described in Section 5.2, aerosol densities in Western Siberia can be high when biomass burning occurs there. Therefore, improving the retrieval accuracy for GOSAT data from the area is an interesting topic. This chapter describes the application of the optimized method to GOSAT data for Western Siberia considering both clear sky conditions and aerosol dense conditions.

### 5.1. Application to Clear Ssky Conditions in Western Siberia and Validation of the Retrieved XCO_2_ Using Ground-Based FTS at Yekaterinburg

At Yekaterinburg (57.038° N and 59.545° E) in Western Siberia, XCO_2_ has been measured using a ground-based Fourier Transform Spectrometer (FTS) by the Ural Federal University. The XCO_2_ data accuracy was reported by Nikita et al. [44]. They are sufficiently accurate to be used for GOSAT data validation. We applied the optimized method to GOSAT data observed near Yekaterinburg during 2010–2014 and retrieved XCO_2_. Then, the retrieved XCO_2_ was validated using data observed at Yekaterinburg. The match-up criterion between GOSAT and ground-based FTS is ±2 × 2 degrees in latitude and longitude, and within a ±1.5 h observational time. In addition, Yekaterinburg is a site of the Aerosol Robotic NETwork (AERONET); AOT has been measured using a sun spectral photometer set. Actually, the AOTs observed at Yekaterinburg for the cases used in the validation were nearly 0.1. Considering the low AOTs and the restriction that ground-based FTS can only take measurements under clear sky conditions, the validation was executed presuming that the atmosphere included no cloud or aerosol.

**Figure 6:**
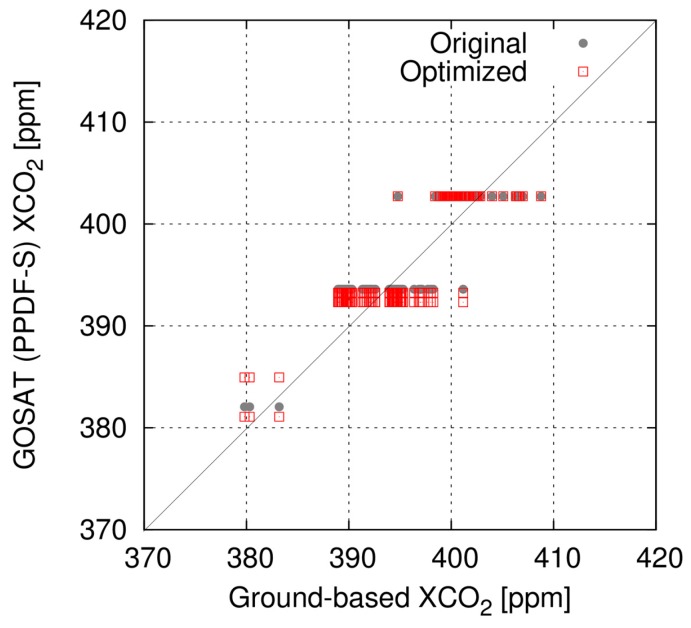
Comparison of XCO_2_ retrieved by the PPDF-S method from the GOSAT data and that observed at Yekaterinburg using ground-based FTS. Gray dots present results of GOSAT data analyzed with the original parameter setting in the PPDF-S method. Red squares are those of the optimized parameter setting.

A comparison of PPDF-S data and ground-based FTS data at Yekaterinburg is presented in Figure 6. The results obtained using the original parameter setting in the PPDF-S method and those obtained using the optimized setting are colored as gray and red, respectively. The numbers of GOSAT data shown in the figure are three and five, respectively, for the original and optimized settings. To represent the retrieval accuracy, the bias and standard deviation of XCO_2_ are calculated by subtracting ground-based data from the GOSAT data. The results show that the bias and its standard deviation of XCO_2_ are, respectively, 0.75 and 0.57 ppm for the original setting, and 0.69 and 1.79 ppm for the optimized data.

### 5.2. Application to Biomass Burning Area in Western Siberia

Biomass burning is a generic term for the burning of forests, grassland, and crop residues. It is known to release aerosols as black carbon and organic carbon into the atmosphere along with greenhouse gases. Western Siberia is a large biomass burning area [45] for which the main land cover type is boreal coniferous forests and croplands. In the southern part of the area, the combustion of spring wheat residues is performed as an activity of farm operations. If the agricultural burning is not managed well, then it can cause uncontrolled wildfires.

**Figure 7:**
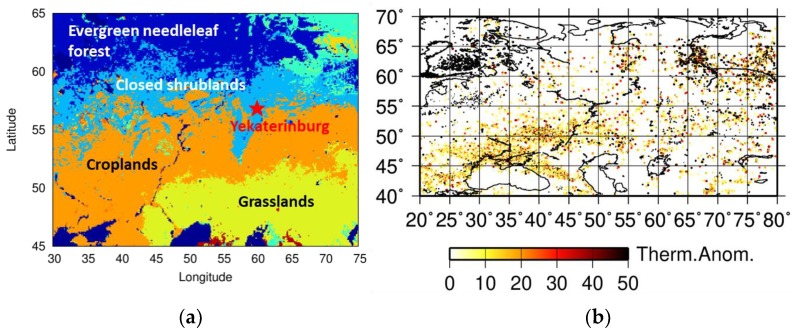
Land type map of the target area and location of the Yekaterinburg observation site (**a**); averaged thermal anomaly (relative units) detected by MODIS during June–August in 2013 (**b**).

To investigate the retrieval accuracy of the optimized PPDF-S method under denser aerosol conditions, we applied the method to the retrieval of XCO_2_ from GOSAT data of the selected Western Siberia area: within 45° N to 65° N latitude and 30° E to 75° E longitude. This area includes an observation site of the Ural Federal University at Yekaterinburg, where XCO_2_ has been measured using ground-based FTS. A land type map of the target area and the location of the Yekaterinburg site are shown in Figure 7a. During the summer of 2013, numerous fires and smoke plumes emitted from biomass burning were detected using a MODerate resolution Imaging Spectroradiometer (MODIS). Furthermore, high AOTs were detected at Yekaterinburg as an AERONET site. For this reason, we specifically undertook the analysis of GOSAT data during the summer (June–August) in 2013 over the Western Siberia area described above. As a measure of biomass burning activity in this area, accumulated counts of thermal anomalies detected by MODIS for the period are shown in Figure 7b.

It is pointed out that the amount of XCO_2_ data retrieved using the original PPDF-S method is less than that of the NIES RA product [32], which is derived using the FP method and only provided for the Research Announcement principal investigators of the GOSAT project. However, after PPDF parameter optimization, the amount of data retrieved using the PPDF-S method increased from the original number for the target area in Western Siberia during the analysis period.

For retrieval analysis of GOSAT measurement data, the atmospheric transmittance of gas species was calculated at the high spectral resolution of 0.01 cm^−1^ using v3.0 ACOS/OCO-2 based on the absorption coefficient (ABSCO) look-up tables (LUTs) provided by the Absorption Coefficient (ABSCO) Team instead of line-by-line (LBL) calculation using the HITRAN database [43]. These LUTs record molecular absorption cross-sections in a three-dimensional space of wavelengths, temperatures, and pressures for O_2_, H_2_O, and CO_2_, including radiative effects of various kinds, such as non-Voigt line shapes, speed dependence, line mixing, and collision-induced absorption [46].

### 5.3. Comparison of Results Retrieved Using Optimized PPDF-S Method and Full Physics Method

**Figure 8:**
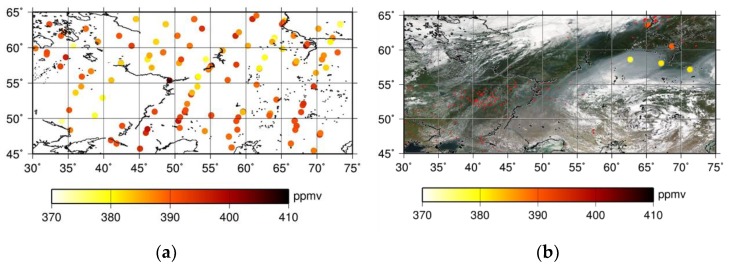
Horizontal distribution of newly retrieved XCO_2_ data analyzed using the optimized method for the target dataset (**a**), and an example of a visible composition image of MODIS for a smoky scene and XCO_2_ values represented by large colored dots observed on 8 August 2013 (**b**). Red points shown in (**b**) are fire positions detected by MODIS/Terra and Aqua on the day.

**Figure 9:**
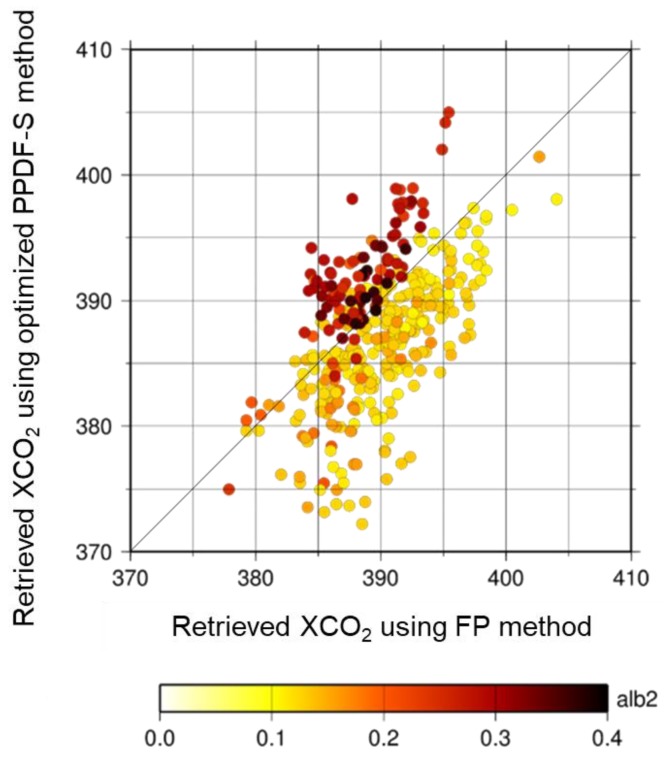
Scatter plot of XCO_2_ analyzed by the optimized PPDF-S method and the FP method. The dot color represents the averaged surface albedo in the spectral band of the GOSAT sensor at 1.6 μm.

The distribution of XCO_2_, which is newly retrieved using an optimized method for the target dataset, is shown in Figure 8a. The amount of data retrieved using the PPDF-S method increased by about 70%. In some cases, XCO_2_ can be retrieved by the optimized method even over smoke from biomass burning. An example of those scenes observed on 10 August 2013 is shown in Figure 8b. A background photograph of the figure is a visible composite image of MODIS. As an overall feature of XCO_2_ distribution, smaller values of XCO_2_ were observed in the northern part of the boundary at about 55° N, with larger values in the southern part. To compare the results of the optimized PPDF-S retrieval and the NIES RA product, the XCO_2_ values analyzed using the PPDF-S method are shown against the NIES RA product in Figure 9. The color scale in the figure represents the averaged surface albedo in the spectral band of the GOSAT sensor at around 1.6 μm. It shows fairly good interrelation among these data, but some data show that the XCO_2_ by the optimized PPDF-S was smaller than the NIES RA product, which ranges from 380 ppm to 370 ppm.

**Figure 10:**
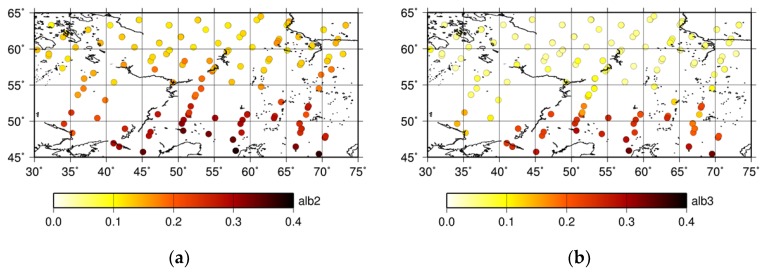
Distribution of the averaged surface albedo in the spectral bands of the GOSAT sensor at around 1.6 μm (**a**) and 2.0 μm (**b**).

**Figure 11:**
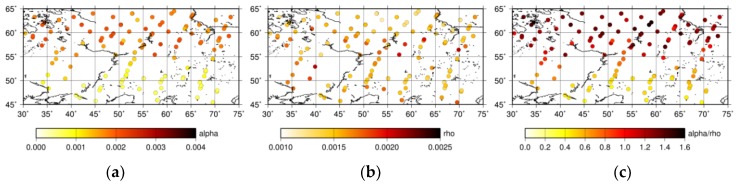
Distribution of the retrieved PPDF parameters: *α_a_* (**a**), *ρ_a_* (**b**), and *α_a_/ρ_a_* (**c**).

Figure 9 shows that the XCO_2_ of the optimized PPDF-S retrieval became large when the albedo at 1.6 μm was large. This result implies that the retrieved XCO_2_ is strongly related to the surface albedo corresponding to the land type in the scene. The distributions of the averaged surface albedo in the GOSAT spectral band at around 1.6 μm and 2.0 μm are shown in Figure 10a,b, respectively. The albedo was evaluated in relation to the retrieval procedure for XCO_2_. In these figures, the surface albedo values for both measurement bands are small, below 0.1, in areas with a latitude higher than 55° N. Larger values were found for lower-latitude areas. The comparison of the results presented in Figure 9; Figure 10 shows that XCO_2_ values retrieved using the PPDF-S method are correlated with the surface albedo. That dependency, which is similar to what Oshchepkov et al. [34] reported, is also investigated through a comparison of the retrieved PPDF parameters. The horizontal distributions of the retrieved *α_a_*, *ρ_a_*, and *α_a_/ρ_a_* are shown in Figure 11a–c, respectively. The *α_a_/ρ_a_* parameter can represent the ratio of the shortening effect for the light-path length caused by light refraction (*α_a_*) to lengthen the effects of the light-path length because of light scattering (*ρ_a_*). The dependence of *α_a_* and *α_a_/ρ_a_* on the surface albedo corresponding to the land surface type is presented in Figure 11. Discussions of these relations among PPDF parameters are presented in Section 6.

### 5.4. Identification of Atmospheric Aerosol Types Using PPDF Parameters

**Figure 12:**
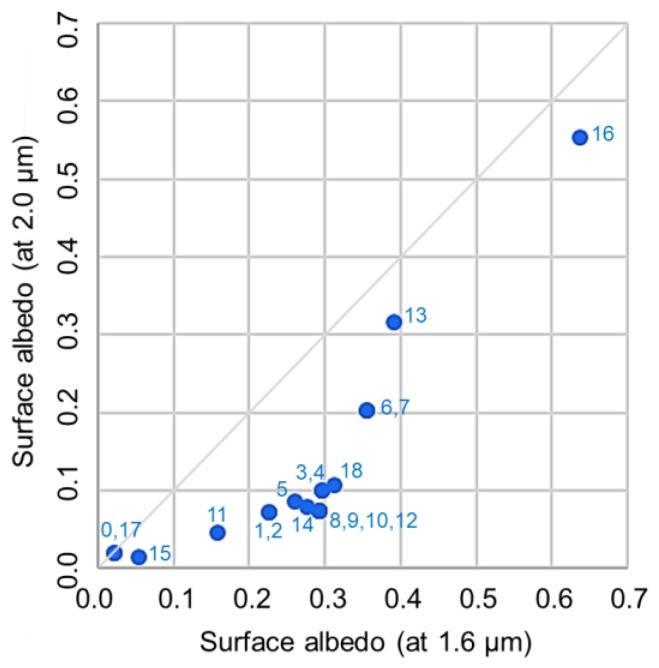
Surface albedo at CO_2_ bands (1.6 μm and 2.0 μm) for each land cover type: 0, water; 1, evergreen needleleaf forest; 2, evergreen broadleaf forest; 3, deciduous needleleaf forest; 4, deciduous broadleaf forest; 5, mixed forest; 6, closed shrublands; 7, open shrublands; 8, woody savanna; 9, savanna; 10, grasslands; 11, permanent wetlands; 12, croplands; 13, urban and built-up areas; 14, cropland/natural; 15, snow and ice; 16, barren or sparsely vegetated areas; 17, water bodies; and 18, tundra. The values are related to the ASTER spectral library.

**Figure 13:**
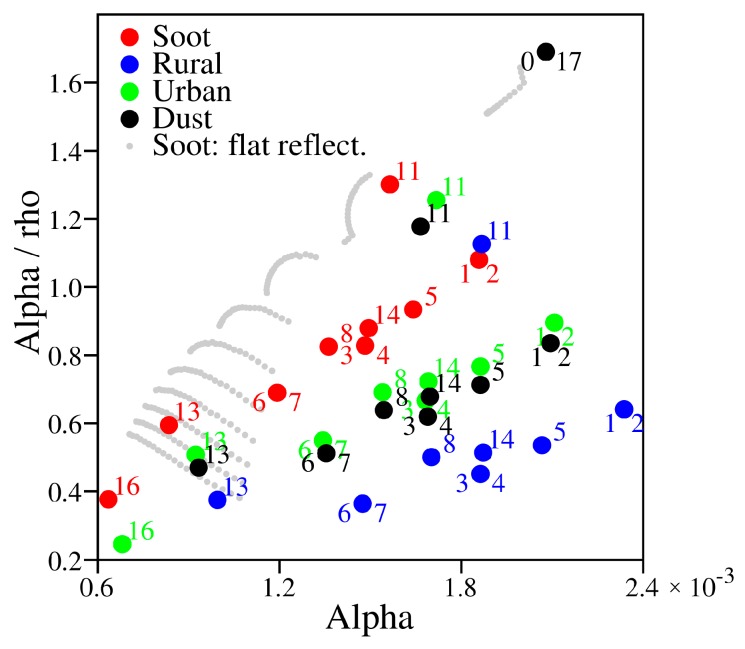
Correlation diagram between *α_a_* and *α_a_/ρ_a_* based on simulations, assuming various surface types: red, Soot; blue, Rural; green, Urban; black, Dust-like. Numbers adjacent to each dot represent the land cover type: 0, water; 1, evergreen needleleaf forest; 2, evergreen broadleaf forest; 3, deciduous needleleaf forest; 4, deciduous broadleaf forest; 5, mixed forest; 6, closed shrublands; 7, open shrublands; 8, woody savanna; 9, savanna; 10, grasslands; 11, permanent wetlands; 12, croplands; 13, urban and built-up areas; 14, cropland/natural; 15, snow and ice; 16, barren or sparsely vegetated areas; 17, water bodies; and 18, tundra. Gray dots show results for Soot obtained from a simulation in which the surface albedo varies from 0.05 to 0.5, but it was the same for all GOSAT spectral bands. For land cover types of ‘0, water’, ‘17, water bodies’, ‘15, snow and ice’, and ‘18, tundra’, values of *α_a_* or *α_a_/ρ_a_* are so large that they exceed the axis range. They are not shown in Figure 13. As the results for ‘9, savanna’, ‘10, grasslands’, and ‘12, croplands’ are overlapping with ’8, woody savanna’, only a label for ‘8’ is plotted on the figure.

**Figure 14:**
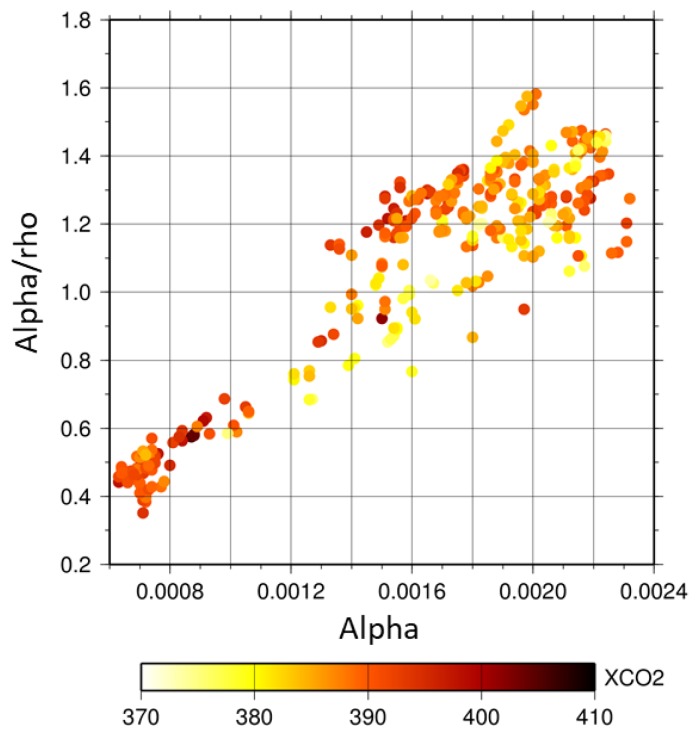
Correlation diagram between *α_a_* and *α_a_/ρ_a_* for GOSAT data observed over the target area in Western Siberia. The color represents retrieved XCO_2_.

As described in Section 5.2, *α_a_/ρ_a_* can be used as a measure to represent the ratio of the shortened light-path length caused by light refraction (*α_a_*) to lengthen the light-path length because of light scattering (*ρ_a_*). These parameters vary, depending on surface reflectance and on the characteristics of aerosols in the atmosphere. Each type of aerosol has its own characteristics of light reflectance/scattering corresponding to its own refractive index and particle size distribution. However, surface reflectance and its wavelength dependence differ among land cover types. We investigated the dependence of *α_a_* and *α_a_/ρ_a_* on surface albedo for each land cover type. In the analysis, the surface albedo was related to the Advanced Spaceborne Thermal Emission and Reflection Radiometer (ASTER) spectral library. Figure 12 shows the surface albedo at CO_2_ bands (1.6 μm and 2.0 μm) for each land cover type. A diagram of the correlation between *α_a_* and *α_a_/ρ_a_* retrieved from simulations is shown in Figure 13. In the simulations, surface albedos of two types were assumed: (1) surface albedo varied as 0.05–0.5, but it was the same for all GOSAT spectral bands changing the AOT from 0.05 to 1.0; and (2) the surface albedo had more realistic values when presuming 19 land cover types related to the ASTER spectral library with a fixed AOT of 0.5. As shown in Figure 13, the result from simulation (1) is depicted by gray plots, but only for the Soot type aerosols as an example. The spread of plots aligned in a row results from the variation of AOT. For simulation (2), results are shown for aerosols of all types. The numbers adjacent to each dot represent the land cover type. The axis range is restricted to the same range as that for the results from the GOSAT data shown in Figure 14. For land cover types of ‘0, water’, ‘17, water bodies’, ‘15, snow and ice’, and ‘18, tundra’, values of *α_a_* or *α_a_/ρ_a_* are so large that they exceed the axis range and they are not shown in Figure 13. As the results for ‘9, savanna’, ‘10, grasslands’, and ‘12, croplands’ are overlapping with ’8, woody savanna’, only a label for ‘8’ is plotted on the figure. We also investigated the correlation between *α_a_* and *α_a_/ρ_a_* for the GOSAT data over the target area in Western Siberia. The result is shown in Figure 14. The color range represents the retrieved XCO_2_. Data are spread along a linear line with simulations for the type of aerosol. Its slope and absolute values are almost identical to those for the Soot-type aerosols in the simulation. The tendency shown in Figure 14 is similar to that for Soot shown in Figure 13, which might imply that Soot aerosols were included in the atmosphere over the target area during the analysis period. The implication is reasonable in light of the fact that the main aerosol types emitted from biomass burning are organic carbon and black carbon that are the same kind of Soot [47]. This also corresponds to the AERONET observation at Yekaterinburg. The observation showed a high value of absorption AOT, which means that atmospheric aerosol types can be Soot. We conclude that the PPDF parameters chart, as shown in Figure 13 and Figure 14, presents the possibility of identifying atmospheric aerosol types.

## 6. Discussion

As described in Section 4.2.1, simulation studies show that the retrieved CO_2_ profile is not vertically uniform, even under a clear sky condition. The non-uniformity might result from the difference in the characteristics of radiative transfer models between Pstar3 used for synthesizing the spectral radiance and the model with PPDF parameters used for simulating a priori radiance. The former is Pstar3, which is based on the Discrete Ordinate Method (DOM), whereas the latter is an original code in which the light-path length modification is represented by the atmospheric transmittance and PPDF parameters. The difference of radiance corresponding to Rayleigh light scattering at Band 1 (0.76 μm) between these two models is about 0.25%.

Simulation studies also show that the XCO_2_ bias for the atmosphere including Dust-like aerosols is very large compared with that for the other types of aerosols (Rural, Urban, and Soot). This result might derive from the strong effects of light scattering by Dust-like aerosols with AOT: they are four times stronger than those of the other aerosols in the main spectral band (1.6 μm) used for CO_2_ retrieval. This might be attributable to the same order of the particle size of Dust-like aerosols as the wavelength. From the retrieval results of the simulation study, we found that the derived XCO_2_ in the first step of the iterative calculation to minimize the cost function ***J*(*x*)** in the PPDF-S retrieval is closest to the true value for Dust-like aerosols. Although the result shows that the possibility of another ***x_a_*** is more appropriate for Dust-like aerosols, adoption of the derived XCO_2_ in the first step as a retrieval result is a good countermeasure against the large XCO_2_ bias for Dust-like aerosols.

**Figure 15:**
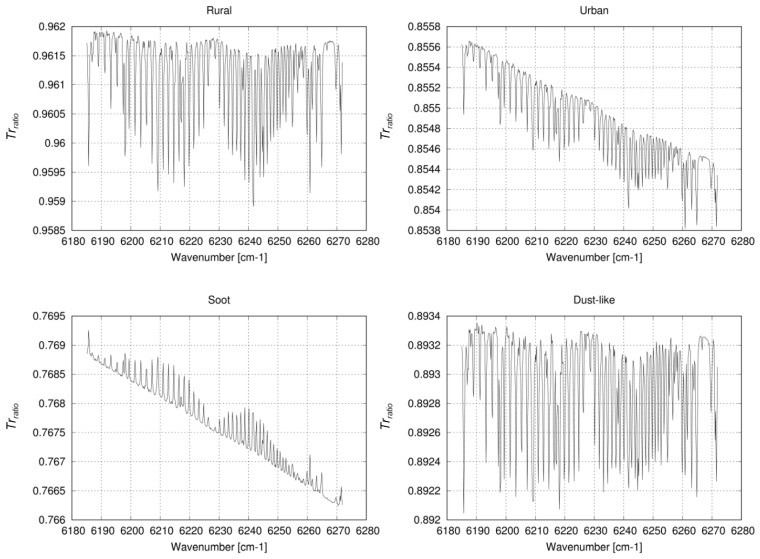
*Tr_ratio_* for Rural, Urban, Soot, and Dust-like with AOT of 0.5 at 0.55 μm for the surface albedo of 0.2, independent of wavelength.

For Soot-type aerosols, XCO_2_ bias is negative under most AOT and surface reflectance conditions. It can result from underestimation of the effects of a shortened light-path length on the PPDF-S retrieval. The effect of light absorption by Soot at 1.6 μm is very strong: the value of the imaginary part of the complex index of refraction of Soot at the wavelength is larger than that of the other aerosols (Rural, Urban, and Dust-like) by about two orders of magnitude. The property also affects the efficiency of light scattering. The size parameter of Soot is about 0.5–1.0 at 1.6 μm. For the size parameter, if the value of the imaginary part of the complex index of refraction is large, then the effect of light scattering will be greater [48]. This means that relative reflection of the aerosol layer (*α_a_*) will be greater. The light scattering also lengthens the light path in the Soot layer by multiple scattering of photons. However, at the same time, the Soot absorbs the photons in the layer. As a result, the light-path length in the layer is shortened. Then, the relative mean path length between the layer and ground surface (*ρ_a_*) will be shorter. Therefore, the total effect of light absorption and scattering by Soot can be effective to make the total light-path length short. The effect is confirmed by the difference of atmospheric transmittance at 1.6 μm between clear sky and the atmosphere including each type of aerosol. The difference is represented by the ratio (*Tr_ratio_*), which is calculated by dividing the transmittance for the atmosphere containing aerosols (*Tr_aerosol_*) by that for clear sky (*Tr_clear_*); *Tr_ratio_* = *Tr_aerosol_*/*Tr_clear_*. The difference between the *Tr_ratio_* on the CO_2_ absorption line and that on the baseline outside the absorption line can be a measure of the magnitude of light-path modification by aerosols. A smaller *Tr_ratio_* on the CO_2_ absorption line represents that light has been absorbed effectively by CO_2_ because of the lengthened light-path length by aerosols. However, larger *Tr_ratio_* on the absorption line represents a shortened light-path length by aerosols. Figure 15 shows *Tr_ratio_* for Rural, Urban, Soot, and Dust-like with AOT of 0.5 at 0.55 μm for the surface albedo of 0.2, which is independent of wavelength. The results show that the *Tr_ratio_* on the absorption line is smaller than that on the baseline for Rural, Urban, and Dust-like; it is larger for Soot. The result shows that the former three aerosol types work to increase the light-path length at the 1.6 μm band, whereas Soot decreases the light-path length. According to the optical effect by Soot described above and the definition of *α_a_* and *ρ_a_*, a larger a priori value of *α_a_* than the original value can represent the shortened light-path length and can reduce the XCO_2_ bias for Soot. A value of 36/20 instead of 1/20 used in the equation for the a priori value of *α_a_* shown in Table 2 for *β_αa_* is most effective at reducing the XCO_2_ bias.

Results for retrieval from GOSAT data measured in Western Siberia show that about 10% of the retrieved XCO_2_ take small values below 380 ppm, as shown in Figure 9. Although the retrieval result accuracy cannot be evaluated because no verification data exist for the target area during the analysis period, the value is extremely small compared with the background level of XCO_2_ for the year in Western Siberia. Therefore, the possibility that the airmass considered in the PPDF-S is larger than the true one can cause the overestimation of XCO_2_. That possibility was investigated. Corresponding to the airmass, we compared the surface pressure for the PPDF-S method and that for the FP method. For the comparison, the a priori value of surface pressure used in the PPDF-S method was used because surface pressure is not derived in the method. However, surface pressure was retrieved along with the RA product of XCO_2_ in the FP method. The retrieved surface pressure was used in the comparison. As a result, the relation of surface pressure between the two methods for the cases in which XCO_2_ retrieved using the PPDF-S method was below 380 ppm was identical to that for other cases in which small XCO_2_ was not retrieved using the PPDF-S method. The a priori value of surface pressure used for the PPDF-S method was not so wrong that it could cause the underestimation of XCO_2_. On the other hand, as described in Section 5.3, Figure 13 and Figure 14 show that the consideration derives from the inference that Soot might be included in the atmosphere. Then, we also considered the possibility that the effect of the shortened light-path length by Soot is underestimated in the PPDF-S retrieval. If the value of 36/20 was used for the equation for the a priori value of α_a_ shown in Table 2 for *β_αa_*, then the value of the retrieved XCO_2_ was increased by about 5 ppm. Because the retrieved XCO_2_ value was more realistic, the identification of Soot from the *α_a_-α_a_/ρ_a_* chart might be appropriate. The AOT values were 0.1–0.4 for the cases of small XCO_2_. Although the value is not so high, the underestimation of XCO_2_ can be caused by negative XCO_2_ biases that can occur even for low AOT, as shown in Figure 5.

We investigated the dependence of retrieved XCO_2_ and PPDF parameters on the altitude of the aerosol layer because light scattering/absorption properties depend on the layer altitude. We executed the PPDF-S retrieval from simulated radiance spectra considering aerosols of four types with the volume mixing ratio at 0–3 km, or at 0–2 km (as shown in Table 1) in Pstar3. Although the difference in XCO_2_ caused by a change in the altitude was as great as 2.05 ppm, the difference in PPDF parameters was small; those for Rural, Urban, Soot, and Dust-like were, respectively, about 5%, 2.5%, 0.01%, and 3%. The difference was so small that one might infer that the dependence of the retrieval results on the aerosol layer altitude is negligible. The result also indicates that the identification of aerosol types using the *α_a_-α_a_/ρ_a_* chart is applicable to various situations of the atmosphere, including aerosols.

**Figure 16:**
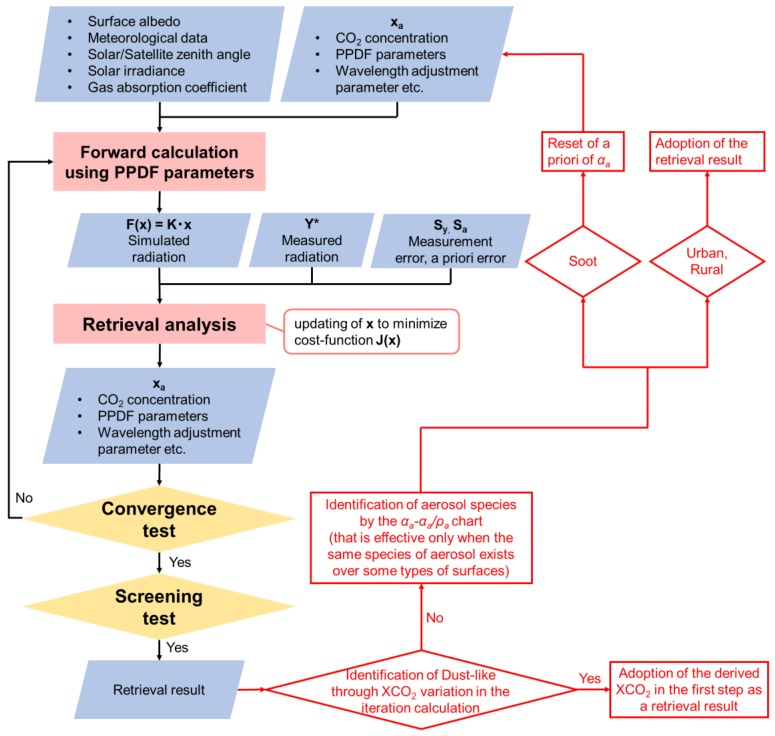
Improved PPDF-S retrieval flowchart.

From the possibility of the identification of aerosol types using the *α_a_-α_a_/ρ_a_* chart and the countermeasures against the large XCO_2_ bias for Dust-like aerosols and against the negative XCO_2_ bias for Soot, a more improved PPDF-S retrieval flowchart can be inferred, as shown in Figure 16. In the figure, the improved part is shown in red letters, in addition to the original flowchart.

## 7. Conclusions

We have improved the PPDF-S retrieval method for XCO_2_ retrieval from GOSAT measurement spectra in SWIR to retrieve XCO_2_ more accurately under dense aerosol conditions. In the improved version, the constraint of a PPDF parameter that represents the light reflectance effect by aerosols (*α_a_*) was optimized based on simulation studies of various types of aerosols and surface albedos, referring to the vertical profile of CO_2_ concentration as a measure of a stable solution. The new method was applied to retrieval analysis of GOSAT data measured in Western Siberia, where biomass burning is very active. By optimizing the PPDF parameter, the retrieval came to be executable, even under smoky conditions, and the total number of retrieved data increased by about 70%. A comparison of the XCO_2_ of the data provided by NIES as an RA product and the XCO_2_ values of newly retrieved data showed no marked inconsistency. The results show that the PPDF parameters retrieved from GOSAT data measured for Western Siberia are consistent with those from the simulations for Soot. The result might imply that Soot aerosols were included in the atmosphere over the target area during the analysis period, which is consistent with real phenomena and ground-based observations. Therefore, we conclude that the PPDF parameters have the possibility of identifying atmospheric aerosol types. The results also show that the effect of shortening the light-path length by Soot can be underestimated by PPDF-S retrieval. According to the simulation studies, the result was solved by setting the a priori value of *α_a_* as larger to represent the shortened light-path length. This countermeasure for Soot was also effective for retrieving more realistic XCO_2_ from GOSAT data for Western Siberia. From these results, we suggest the use of a further improved algorithm after the atmospheric aerosol type can be identified. The algorithm is anticipated for application to the analysis of carbon monoxide (CO), which is newly observed by the successor of GOSAT: GOSAT-2. The results of simultaneous observations and analyses of CO_2_, CO, and aerosols using the method are expected to contribute to a better capability of distinguishing anthropogenic CO_2_ emissions from the total emissions of urban areas or biomass burning regions where gases and aerosols are released simultaneously.

## Figures and Tables

**Table 1:** Parameter settings and datasets for simulating spectral radiance

Parameter	Details
Multiple scattering radiative transfer model	Polarization System for Transfer of Atmospheric Radiation3 (Pstar3) [40]
Solar Irradiance Model	Band 1: Kurucz’s model [41]/Bands 2, 3: Toon’s model [42]
Zenith angle	Solar: 30°/Satellite: 0°
Surface albedo	0.05–0.50 (Bands 1, 2, 3)
Surface pressure	Grid Pointed Value (GPV) data of middle latitude summer from Japan Meteorological Agency (JMA)
Temperature and pressure profile	
Water vapor (H_2_O) profile	
Carbon dioxide (CO_2_) profile	390 ppm in all layers
Aerosol types	Dust-like Urban, Rural, Soot (volume mixing ratio is given at 0–2 km)
Aerosol Optical Thickness (AOT)	0.05–1.0
Gas absorption	Line-By-Line (LBL) calculation using HIgh resolution TRANsmission molecular absorption database (HITRAN) 2004 [43]

**Table 2:** A priori and variance of respective retrieval parameters.

Parameter	A priori (*x_a_*)	Variance (*σ_a_*)
CO_2_	385 ppm in all layers	$S_{a}^{i,j}={\left(S_{a}^{i,i}\cdot S_{a}^{j,j}\right)}^{\frac{1}{2}}\exp\left[-0.5\left|\ln\left(p_{i}/p_{j}\right)\right|\right],$ where *σ_a_^i,i^* = 6 ppm and *p_i_* is pressure at the *i*th level.
*h_r_*	5 km	0.001 km
*β_αr_* ^1^	$\sqrt{-\ln\left(\alpha_{r,Band1}\right)}\times \left(\Gamma_{i}/\Gamma_{1}\right)\approx 2,$ where *Γ_i_* is surface albedo at Band *i* (*i* = 1, 2, 3).	0.01
*β_ρr_* ^2^	1	0.01
*β_γr_* ^3^	3	0.002
*h_a_*	5 km	0.5 km
*β_αa_* ^1^	$\sqrt{-\ln\left(\alpha_{r,Band1}/20\right)}\times \left(\Gamma_{i}/\Gamma_{1}\right)\approx 3$	0.1
*β_ρa_* ^2^	1	$0.1\times \sqrt{10}$ (for Gain H ^4^), $0.1\times \sqrt{0.5}$ (for Gain M ^5^)
*β_γa_* ^3^	3	$0.1\times \sqrt{10}$ (for Gain H ^4^), $0.1\times \sqrt{0.5}$ (for Gain M ^5^)

^1^$\alpha =\exp\left[-\beta_{\alpha }^{2}\right]\times ScaleFactor_{\alpha }$, ^2^$\rho =\frac{\rho_{r,Band1}}{\alpha_{r,Band1}}\times \beta_{\rho }\times ScaleFactor_{\rho }$, ^3^$\gamma =\exp\left[-\beta_{\gamma }^{2}\right]\times ScaleFactor_{\gamma }$, (*ScaleFactor_x_*: Adjustment parameter for representing the dependence of parameter *x* on wavelength). ^4^ Gain H: Case of ‘High’ gain setting of GOSAT sensor. ^5^ Gain M: Case of ‘Middle’ gain setting of GOSAT sensor.
